# Inference of Gene-Phenotype Associations via Protein-Protein Interaction and Orthology

**DOI:** 10.1371/journal.pone.0077478

**Published:** 2013-10-23

**Authors:** Panwen Wang, Wing-Fu Lai, Mulin Jun Li, Feng Xu, Hari Krishna Yalamanchili, Robin Lovell-Badge, Junwen Wang

**Affiliations:** 1 Department of Biochemistry, LKS Faculty of Medicine, The University of Hong Kong, Hong Kong SAR, China; 2 Shenzhen Institute of Research and Innovation, The University of Hong Kong, Shenzhen, China; 3 Division of Developmental Genetics, MRC National Institute for Medical Research, The Ridgeway, Mill Hill, London, United Kingdom; 4 Centre for Genomic Sciences, LKS Faculty of Medicine, The University of Hong Kong, Hong Kong SAR, China; University of Southern California, United States of America

## Abstract

One of the fundamental goals of genetics is to understand gene functions and their associated phenotypes. To achieve this goal, in this study we developed a computational algorithm that uses orthology and protein-protein interaction information to infer gene-phenotype associations for multiple species. Furthermore, we developed a web server that provides genome-wide phenotype inference for six species: fly, human, mouse, worm, yeast, and zebrafish. We evaluated our inference method by comparing the inferred results with known gene-phenotype associations. The high Area Under the Curve values suggest a significant performance of our method. By applying our method to two human representative diseases, Type 2 Diabetes and Breast Cancer, we demonstrated that our method is able to identify related Gene Ontology terms and Kyoto Encyclopedia of Genes and Genomes pathways. The web server can be used to infer functions and putative phenotypes of a gene along with the candidate genes of a phenotype, and thus aids in disease candidate gene discovery. Our web server is available at http://jjwanglab.org/PhenoPPIOrth.

## Introduction

Phenotypes denote the observable physical or biological traits of an organism. Understanding the relations between genes and gene functions (or related phenotypes) is one of the main objectives of genetics in the post-genome era [1] [2] [3]. With the advent of OMICS techniques, the number of uncovered gene-phenotype associations has increased significantly over the last several decades. However, the number of genes with identified phenotypes has not been able to reach the genomic scale yet, due to some technical challenges such as the multi-functionality of genes and heterogeneity of diseases [4]–[6]. At this moment, various types of proteomic and/or genomic data (such as protein-protein interaction (PPI) data [6]–[12], sequence data [13], [14] and function annotations [15]–[19]) have been used to identify gene-phenotype associations. Previous studies showed that products of different genes tend to physically interact with each other if these genes are involved in causation of similar disorders [20], [21]. Similar phenotypes are determined by genes with related functions, too [22]. Researchers used this information to predict phenotypes by the interactome [6], [8] or by the topology of the PPI network [7]. Moreover, sequence information, together with function annotations, has been used to prioritize candidate gene-phenotype associations. For example, the features of sequence data were used to build a model, which was then trained by the function annotations [13], [14], [23]. Researchers also employed machine learning approaches and function annotations to construct models[16] [24] [14].

The cross-species information has been frequently used to study human diseases and to identify human disease genes [17], [25]–[30]. Chen *et al* applied phenotypes of mouse to improve prioritization of human disease causal genes. The prioritization was implemented based on high-throughput genome-wide data [28]. Researchers have also studied human orthologs in model organisms to explore the relationship of human phenotypes and diseases [17], [29], [30]. The “orthology-function conjecture” - orthologs tend to retain the functions from ancestors - was widely applied to annotate gene functions [31], [32], though it has been criticized to be “weak” by some researchers [33], [34]. Nevertheless, function transfer among orthologs is still supported [35], and a domain-based filter could improve its reliability [36]. As the orthology-function indicates, a gene and its orthologs may have similar functions, if they have not experienced much duplication during evolution [37]. For instance, CLCN5 is reported to be associated with several phenotypes, such as *proteinuria*, *hypercalciuric nephrocalcinosis* (OMIM: 308990) [38], [39], *dent disease* (OMIM: 300009) [40] and *nephrolithiasis, type I* (OMIM: 310468) [41] in humans; while its orthologous gene Clcn5 is known to be responsible for *increased urine protein level* (MP: 0002962), *abnormal renal protein reabsorption* (MP: 0011445), *abnormal tooth development* (MP: 0000116) and *nephrocalcinosis* (MP: 0003197) in mouse. The two orthologous genes share the same domains and have a sequence identity of 0.97. Because the close relationship between sequence similarity and phenotype similarity, orthology data has a potential to be used for gene-phenotype association identification.

In addition, gene-phenotype associations have been studied in species other than humans. PhenomeNET [17] is a cross-species phenotype network using function annotations to infer gene-phenotype associations of different organisms. Nicole *et al* tried to extend the human diseases to animal models using an ontology-base method [42]. Over the years, a number of integrative databases have emerged. They have collected known gene-phenotype associations of different species by function annotations [43], [44]. However, these resources did not take both the PPI and orthology information simultaneously. In this article, we used PhenomeNET to connect cross-species phenotypes, and proposed a method to integrate both PPI and orthology information to perform gene-phenotype association inference for six species: fly (*drosophila melanogaster*), human (*homo sapiens*), mouse (*mus musculus*), worm (*caenorhabditis elegans*), yeast (*saccharomyces cerevisiae*), and zebrafish (*danio rerio*). The results were evaluated with the top 100 genes that have the highest number of phenotypes identified. We drew the ROC curves and achieved the AUC values of 0.805, 0.825, 0.740, 0.780, 0.861 and 0.755 for fly, human, mouse, worm, yeast and zebrafish, respectively. Further, we investigated the inferred genes of two human representative diseases, Diabetes Mellitus type 2 (OMIM: 125853) and Breast Cancer (OMIM: 114480), and performed the statistical analysis with Gene Ontology (GO) [45] and with the Kyoto Encyclopedia of Genes and Genomes (KEGG) pathways [46]. Related GO terms and pathways enriched with each disease were observed. We have implemented this method as an online resource, which is now publicly available at http://jjwanglab.org/PhenoPPIOrth. Our online resource can fetch the candidate genes of a given phenotype (or the potential phenotypes of a particular gene) and display them accordingly in an intuitive manner.

## Materials and Methods

### Data Preparation

#### Gene and protein data

Gene and protein data of the six species were obtained from BIOMART of Ensembl (http://www.ensembl.org/biomart/martview). As we focused mainly on PPIs and orthologous proteins, the genes retrieved were restricted to the protein-coding genes. The corresponding Ensembl Protein ID was considered because it would be cross-linked to the PPI and orthology data.

#### PPI and orthology data

The PPI and orthology data were retrieved from the online database resource, Search Tool for the Retrieval of Interacting Genes (STRING) database [47]. The experimentally validated PPI in Human Protein Reference Database (HPRD) [48] were also incorporated by assigning a solid high score of 0.9. The combined score was calculated using the same strategy of STRING [47]. Each interaction was assigned by a combined score of various sources, indicating the reliability of the interaction. Since the majority of interactions in STRING were derived from computations based on prediction algorithms or interolog inference, we abandoned the interactions with a combined score less than 0.5. We also obtained orthologous proteins data from the STRING database, and scanned the domains by PfamScan[49] for further domain composition calculation (see Prioritization of gene-phenotype associations).

#### Phenotypes and known gene-phenotype associations

The majority of the phenotypes were downloaded from the Open Biological and Biomedical Ontologies (http://www.obofoundry.org/). Known gene-phenotype associations were retrieved from the database of each corresponding species (Table S1). For humans, we incorporated two databases, the Human Phenotype Ontology (HPO)[50] and the Online Mendelian Inheritance in Man (OMIM), into our database [51], and connected OMIM to HPO by annotations from http://www.human-phenotype-ontology.org.

#### PhenomeNET

PhenomeNET is a cross-species phenotype network, in which the similarity between the nodes was calculated based on the information content of ontology terms [17]. We employed the information of the node pairs with a similarity score ≥0.5. With this network, the phenotypes from different species are available to be compared. PhenomeNET is available at http://phenomebrowser.net/availability.html.

### Prioritization of gene-phenotype associations

**Figure 1:**
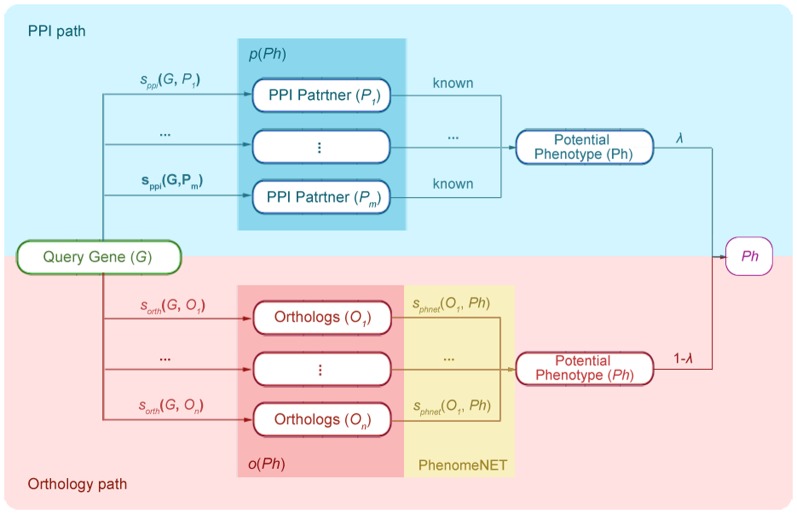
Workflow to infer phenotypes for a query gene. A potential phenotype *Ph* of query gene *G* could be inferred from both PPI and orthology paths, which are marked as light blue and light red pane, respectively. *s_ppi_*(*G*, *P_i_*) refers to the PPI score derived from STRING database, and *s_orth_*(*G*, *O_i_*) represents the domain similarity of the products of orthologous genes. The gene sets *p*(*Ph*) and *o*(*Ph*) in the blue and red box stand for the PPI partners and orthologs of query gene *G*, respectively. The PPI partners are known to be associated with *Ph*. Orthologs are associated with *Ph* through phenomeNET, with the phenotype similarity *s_phnet_*(*O_i_*, *Ph*) (yellow box). These two scores are combined to obtain the final score of the gene-phenotype pair (*G*, *Ph*) after a weightλhas been assigned to the PPI path.

Figure 1 describes the workflow of our method. A phenotype *Ph* could be inferred to be associated with a gene *G* via one or multiple PPI path(s) and/or orthology path(s). *Ph* is derived from PPI if *Ph* is reported to be associated with gene *P*, which is an interactive partner of *G*. Similarly, *Ph* is inferred from orthologs if *Ph* is associated with gene *O*, which is identified as an ortholog of *G*,through PhenomeNET, of the other five species. Either a phenotype is involved in the PPI or in the orthology path of a gene; we regarded it as a potential phenotype of that gene, and a gene could have multiple potential phenotypes. Then we tried to prioritize the gene-phenotype associations by giving scores to inferences from PPI and from orthology paths.

For the PPI path (PPI path in Figure 1), all the interactive partners of *G* were taken into account. These partners have to be associated with the phenotype to be prioritized. We then obtained the raw PPI score for a gene-phenotype pair (*G*-*Ph*) as follows: 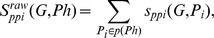
where *p*(*Ph*) refers to all genes that are known to be associated with *Ph*. *P_i_* is one of them, and *s_ppi_*(*G, P_i_*) is the PPI score between the products of *G* and *P_i_* derived from the STRING database.

For the orthology path (Orthology path in Figure 1), similarly, we calculated the raw orthology score for *G*-*Ph* first by considering all genes that are: 1) found to connect to *Ph* in PhenomeNET and 2) identified as an orthologous gene of *G*. The raw orthology score for *G*-*Ph* was calculated as follows: 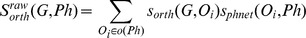
where *o*(*Ph*) refers to all orthologs of G that are related to *Ph* in PhenomeNET. *O_i_* is one of them. *s_orth_*(*G*,*O*) refers to the domain similarity of *G* and *O_i_*. *s_phnet_*(*O_i_,Ph*) stands for the pre-computed cross-species gene-phenotype score from PhenomeNET.

For the domain similarity, we first scanned the protein domains by PfamScan, and then obtained a vector with domains and their counts of each protein. The domain similarity was calculated by the cosine similarity as follows: 
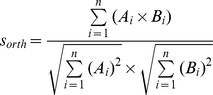
where *A_i_* and *B_i_* represent the number of the same domain of two proteins, respectively, and *n* represents the total number of unique domains of the two proteins scanned.

Finally, the raw score for a gene-phenotype pair is composed of the above two scores with the pre-defined or user-assigned weight (*λ*) of the PPI score as presented in the following equation: 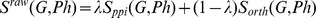
in which *S_ppi_*(*G,G_ph_*) represents the score which was normalized by being divided by the maximal raw score of potential phenotypes of G inferred from PPI; whereas *S_orth_*(*G,Ph*) represents the score which was normalized by being divided by the maximal raw score of potential phenotypes of *G* inferred from orthology paths. We then normalized *S^raw^*(*G,Ph*) for *G*-*Ph* pairs and used this score to prioritize the gene-phenotype associations: 
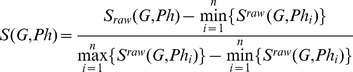
where *Ph_i_* represents any of the potential phenotypes of *G* inferred from either PPI or orthology paths, whereas *n* is the total number of potential phenotypes of *G* inferred from both PPI and orthology paths.

### Description of the Web Server

The web server was built with PHP language (http://php.net) and the open source database MySQL (http://www.mysql.com) on the server side, and with a user-friendly interface on the client side. Users can check all the contents of the data in just one browser window, with different categories of information in different tabular views. The uniform resource identifier of a tab is recorded in the form of browser cookies once the tab opens. It can be re-checked in the ‘History’ tab. We provide both simple search and advanced search options for users to access the web server. Using the simple search option, users can simply type a keyword or an identifier of genes, proteins, or phenotypes into the search box on the upper right, which will persist through the whole session. Alternatively, users can open the ‘Advanced Search’ tab to perform advanced search, which offers a list of suggested keywords when species, entries and attributes are specified.

**Figure 2:**
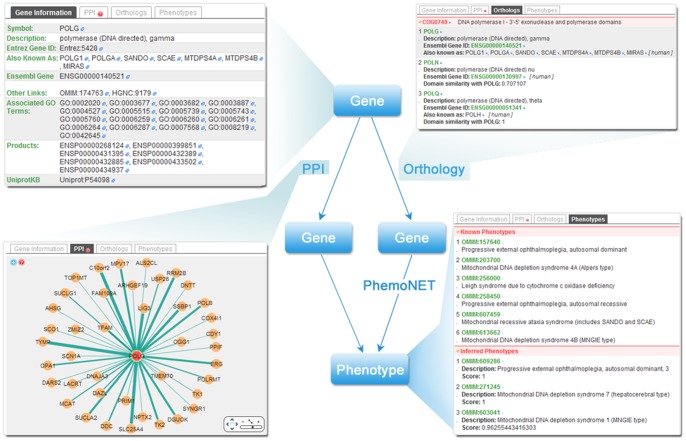
Main workflow and contents of the web Server. Phenotypes of a gene are inferred via PPI paths and orthology paths. All elements in the web server are well annotated. Each element offers a cross-reference link that links to its original source or to the NCBI database. Genes are described by their names, descriptions, Entrez Gene ID, synonyms, Ensembl Gene IDs and other cross-reference links. Their products are identified by their Ensembl Protein IDs and UninprotKB Accessions. PPI information is presented by a network visualization tool, Cytoscape Web[61]. Orthologs are listed and grouped by the orthologous groups. The known phenotypes are listed above the inferred ones, which are sorted in a descendant order by the score.

Upon submission of the keywords by the user, the server will list all the related records including s their orthologs and phenotypes in all six species. Proteins will be automatically connected to their genes as part of the gene information. Figure 2 depicts the scenario that a gene is selected as the entry point to the server. The scores of the potential phenotypes of this gene are calculated as previously described. For each phenotype in the inferred list, the inferring paths are displayed on the right, which can be retrieved and located in the ‘PPI’ and ‘Orthologs’ tabs. It is also possible for users to use the phenotypes as entry points to infer their candidate genes. In this way, the results are fetched from the pre-computed gene-phenotype pairs. The inferring paths will still be shown but will not be locatable (Figure S1).

Although we have pre-set the parameter *λ* (details were discussed in the “Results” section) for each species, users could assign a different weight if desired. Besides *λ*, in the ‘Settings’ tab, users can turn on the option to indicate whether they would like to use only the experimental PPI data, rather than the PPI data obtained by both experimental and computational methods, for inference.

## Results

### Determination of the parameter*λ*

**Figure 3:**
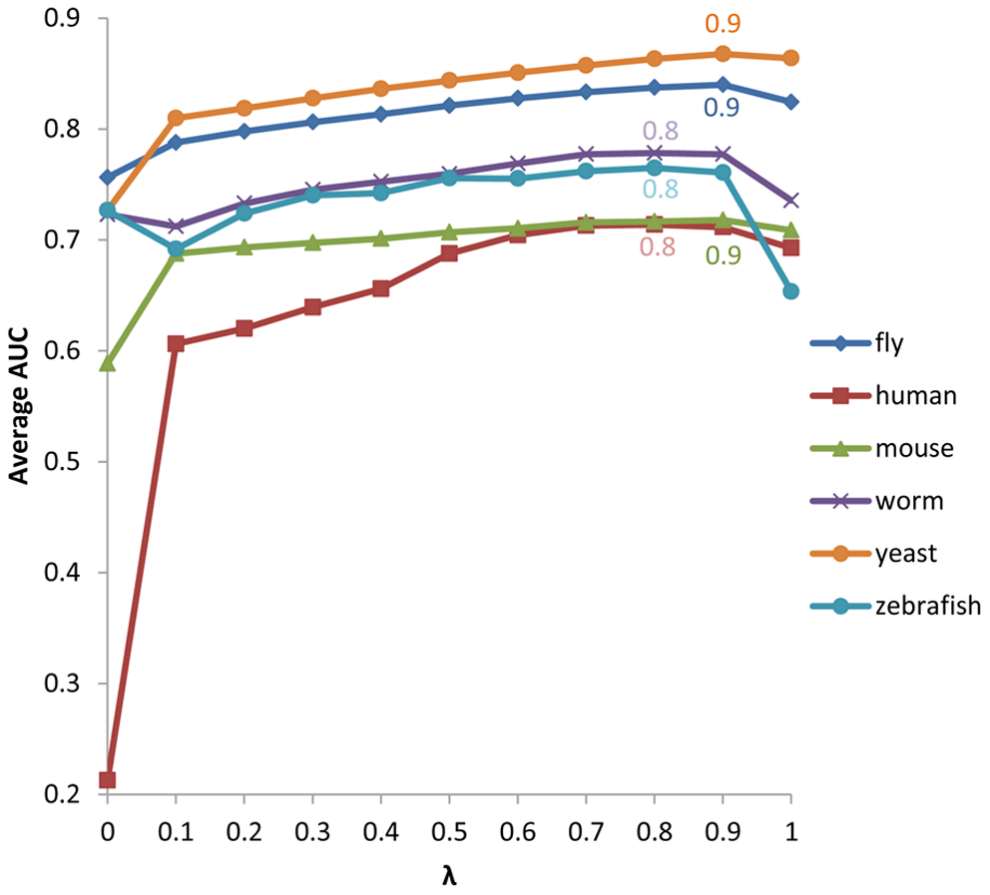
The average AUC values of the inference are affected by the parameter *λ*. The average AUC values were calculated for each species based on the top 100 genes having the highest number of identified phenotypes. ***λ*** defined for fly, human, mouse, worm, yeast and zebrafish are 0.9, 0.8, 0.9, 0.8, 0.9 and 0.8, respectively, which present the maximal average AUC values and are marked in the consensus color as the series of each species.

According to our scoring function, for a given gene, *λ* is the only parameter that could be pre-set to affect the ranking list of candidate phenotypes, as the PPI and orthology data have already been determined in the database. We used genes with phenotypes annotated and with both PPI and orthology data to determine *λ* for each species. We counted the number of annotated phenotypes of each gene and ranked them in a descendent order. Since many genes have unreported phenotypes, here we only used the top 100 genes to determine *λ*. We then drew a receiver operator characteristic (ROC) curve (data not shown) and calculated the area under the curve (AUC) [52] for each chosen gene. If an inferred phenotype agrees with the known phenotype, it is regarded as a true positive, otherwise as a false positive. On the other hand, if a phenotype below the cutoff agrees with the known phenotype, it is regarded as a false negative, otherwise a true negative. We changed the *λ* from 0 to 1 with step of 0.1 and calculated AUCs for each of the 100 genes for each species. The means of these AUCs were calculated and their relationships with *λ* were shown in Figure 3. The value of the parameter*λ* of a species was taken as the one that led to the maximal average AUC for the 100 genes. The defined*λ*would be used to evaluate the results for each species, as well as the suggested parameters in the web server. Figure 3 also indicates that both PPI and orthology information could contribute to the inference of gene-phenotype associations. Taking humans as an example, we defined *λ* as 0.8, which presents 0.714 as the average AUC, whereas when *λ* equals 0 (which implied that no PPI information has been involved in the inference), its AUC value is 0.213. The AUC value turns to 0.693 when *λ* is set to 1, meaning that only PPI is employed to identify the associations. The data for all species could be checked in Table S2.

### Evaluation with Known Gene-Phenotype Associations

**Figure 4:**
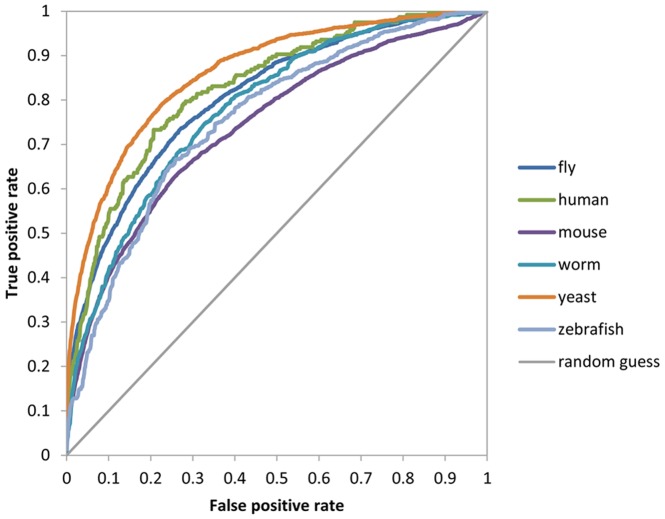
ROC curves for predicting gene-phenotype pairs for each species. The diagonal line stands for a random guess. The AUC value is expected to be 0.5. AUCs for fly, human, mouse, worm, yeast and zebrafish are 0. 805, 0.825, 0.740, 0.780, 0.861 and 0.755, respectively.

We used *λ* to predict phenotypes for the top 100 genes ranked by the number of known phenotypes and with phenotypes inferable by our algorithm. For each species, we pooled phenotypes of all 100 genes and drew the ROC curves. The gene-phenotype pairs above the cutoff agreeing with the known pairs are regarded as true positives, otherwise as false positives. If the gene-phenotype pairs below the cutoff agreeing with the known pairs, they are false negatives; otherwise, true negatives. So the sensitivity (true positive rate) indicates the rate of true positives above the cutoff, and the false positive rate represents the rate of false positives below the cutoff. As shown in Figure 4, the AUC values are significantly higher than those of random guess.

To further demonstrate the reliability of our method, for each species we defined the best cutoff by applying the descending diagonal intersection criterion. In another word, the intersection point of the descending diagonal and the ROC curve was selected as the best cutoff point, which achieves the same cost of true positive rate increase and false positive rate decrease. We used the corresponding cutoff for each species, and randomly picked the same number of phenotypes from the phenome to substitute the positives. The randomization was performed for 100 times. We calculated the Matthews Correlation Coefficient (MCC) for both inferred and randomized results using the following equation: 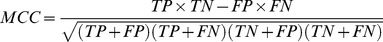
where *TP*, *TN*, *FP* and *FN* refer to the true positives, true negatives, false positives and false negatives defined above, respectively. MCC returns a value between −1 and +1. +1 means a perfect prediction. 0 means that the prediction is no better than a random guess. −1 indicates that the prediction totally disagrees with the observation. The results were shown in Figure S2. MCCs of randomization are close to 0 and are significantly lower than the corresponding values of our prediction.

### Evaluation with Gene Ontology and KEGG Pathway

Gene ontology (GO) [45] is an annotation in common language, which presents the conserved functions of genes or their products. It has three categories: biological process, molecular function and cellular component, referring to the biological objectives, biochemical activities and active locations of genes (or their products), respectively. If genes or their products share the same biological properties, a unified GO term was assigned. The Kyoto Encyclopedia of Genes and Genomes (KEGG) pathway database [46] connects interactions, reactions and relations of molecules or genes if they share the same metabolic mechanism, genetic information processing, environmental processing, cellular processes, organismal systems or human diseases, or even if they have some similarities in structures for drug development. Both databases have been applied to perform the evaluation of the inferred genes of a disease, by performing the hypergeometric test between the inferred gene set of the disease and the gene set of each GO term (or pathway) to check how the enriched GO terms (or pathways) are related to the disease. The GO and pathway gene set data were downloaded from the Molecular Signatures Database (MSigDB v4.0) (http://www.broadinstitute.org/gsea/msigdb/index.jsp)[53]. When conducting the analysis with GO gene sets, the number of genes in the inferred gene set was limited to 90, which is the average gene number of gene sets for all GO terms. This number is 69 for the pathways. Human diseases Diabetes Mellitus type 2 (also known as noninsulin-dependent diabetes mellitus, NIDDM) (OMIM:125853) and Breast Cancer (BC) (OMIM:114480) were chosen, and the top 5 entries having the most enriched GO terms and pathways are listed in Table 1 and Table 2, respectively. Similarly we applied this approach to the mouse phenotype Insulin Resistance (MP:0005331) and GO. The results are shown in Table S3.

**Table 1:** Most enriched GO terms for Breast Cancer and Diabetes Mellitus type 2.

Disease	P value	GO term
Diabetes Mellitus type 2	1.03E-06	Insulin receptor signaling pathway (GO:0008286)
	3.77E-04	Transmembrane receptor protein tyrosine kinase signaling pathway (GO:0007169)
	4.09E-04	Phosphotransferase activity alcohol group as acceptor (GO:0016773)
	7.22E-04	Kinase activity (GO:0016301)
	9.08E-04	Sterol binding (GO:0032934)
Breast Cancer	2.03E-07	Apoptotic process (GO:0006915)
	2.08E-07	Programmed cell death (GO:0012501)
	6.90E-07	Cell cycle (GO:0007049)
	8.66E-07	Regulation of cell cycle (GO:0051726)
	1.40E-06	Regulation of apoptosis (GO:0042981)

**Table 2:** Most enriched KEGG pathways for Breast Cancer and Diabetes Mellitus type 2.

Disease	P value	KEGG pathway
Diabetes Mellitus type 2	4.18E-06	Insulin signaling pathway (hsa04910)
	1.37E-05	Type II diabetes mellitus (hsa04930)
	1.57E-05	Riboflavin metabolism (hsa00740)
	6.27E-05	Maturity onset diabetes of the young (hsa04950)
	6.56E-05	Renal cell carcinoma (hsa05211)
Breast Cancer	3.93E-04	Oocyte meiosis (hsa04114)
	2.40E-03	Apoptosis (hsa04210)
	8.00E-03	Nucleotide excision repair (hsa03420)
	1.13E-02	Amyotrophic lateral sclerosis (ALS) (hsa05014)
	1.57E-02	Calcium signaling pathway (hsa04020)

Glucose uptake is induced by the activation of insulin receptor. The cells are incapable of taking up glucose partly due to a decrease in insulin receptor signaling (involving in *Insulin receptor signaling pathway* (GO:0008286)), resulting in NIDDM. The insulin receptor is also a transmembrane receptor, which can be activated by insulin. So this process is also related to *Transmembrane receptor protein tyrosine kinase signaling pathway* (GO:0007169), a parent term of GO:0008286 [54]. The molecular function GO term *Kinase activity* (GO:0016301) is related since this receptor belongs to the tyrosine kinase receptors class [55]. For enriched pathways of NIDDM, as stated previously, the insulin receptor can be activated by insulin. As a result, the *Insulin signaling pathway* (hsa04910) can also lead to NIDDM. The pathway *Type II diabetes mellitus* (hsa04930) is directly associated with NIDDM, and *maturity onset diabetes of the young* (MODY) (hsa04950), a monogenic form of NIDDM, is suffered by 2–5% diabetic patients [56]. It is caused by heterozygous mutations of multiple transcription factors, including HNF1alpha (MODY3, HNF1A)[57] and PDX1 (MODY4)[58], which are two of the inferred genes of NIDDM and also are present in the gene set of pathway hsa04950.

The formation of BC is similar to that of other cancers. The cells are unable to stop division and cannot be delivered to where they belong. In these cells, apoptosis (*Apoptotic process* (GO:0006915)) is disrupted. This process is also associated with *Programmed cell death* (GO:0012501), a parent term of GO:0006915, and *Regulation of apoptosis* (GO:0042981). Accordingly, the pathway *Apoptosis* (hsa04210) is also related. *Nucleotide excision repair* (hsa03420) is considered a relevant pathway since researchers [59] have revealed that lack of DNA repair capacity can be a risk factor of BC. *Calcium signaling pathway* (hsa04020) may also be associated with BC as cellular calcium signals have been involved in regulating apoptotic pathways and inducing apoptosis [60].

## Discussion

Gene-phenotype association identification is one of the common goals of biological studies. However, difficulties and challenges exist in both computational and experimental approaches. Researchers have applied sequence data, PPI data and function annotations to identify gene-phenotype associations, but together with comparative sequence information, such as orthology, has not been taken into account thus far. The PhenomeNET, a cross-species phenotype network, is applied here to connect phenotypes among different species. Subsequently, we employed orthology, as well as PPI information, to perform gene-phenotype identification. We used a simple linear function to combine the two types of information, and normalized the PPI and orthology items before they were joined into the final score, which was normalized by being divided by the maximal score of the phenotypes of a given gene. In this manner, the phenotypes with the score consisting of only PPI or only orthology item, have a chance to stay at the top of the ranked list, which may lower the confidence of evidence by both of the items. However, it is expected that phenotypes inferred by both PPI and orthology would have a higher priority. Introducing other weighting strategies to enhance these potential phenotypes would worth a trial.

Our method is flexible to encompass data of more species. If there are sufficient data of a new species, including PPI, orthology, and function annotation that are used to extend the PhenomeNET, the species is ready to be incorporated into our database. The new species will also benefit and be benefited from other species as more cross-species information is joined. We also observed that both PPI and orthology information could enhance the ability to identify the phenotypes of genes. PPI and orthology data sets may assist identification of gene-phenotype associations cooperatively if both of them are available for a gene-phenotype pair, or complementary to each other if one of them is found.

The ability of our method to identify the potential phenotypes of genes offers more reference to our understanding of gene functions. The functions of some genes may not be fully revealed or verified experimentally. The potential phenotypes would provide biologists guidance to study the genes. Additionally, inferring the candidate genes of phenotypes, especially diseases, helps to uncover the mechanisms of diseases. Identifying the products of candidate disease genes as new targets can facilitate drug development as well.

## Supporting Information

Figure S1 **Workflow and contents when accessing the web server from phenotypes.** The candidate genes of the query phenotypes are retrieved from the pre-computed gene-phenotype associations. (TIF).
(TIF)

Figure S2 **MCC of our prediction and randomization for each species.** The MCC values for the prediction and 100-time randomization. (TIF).
(TIF)

Table S1 **Phenotype and known gene-phenotype association sources.** The sources from which the phenotype data and known gene-phenotype associations were retrieved are listed. (TXT).
(DOCX)

Table S2 The average AUC values of different *λ* for the six species. (TXT).
(DOCX)

Table S3 The most enriched GO terms for mouse phenotype insulin resistance (MP: 0005331). (TXT).
(DOCX)
