# Supplementary figures and images for: Inference of Gene-Phenotype Associations via Protein-Protein Interaction and Orthology
Source: PLoS One. 2013 Oct 23;8(10):e77478. doi: 10.1371/journal.pone.0077478 (PMC3806783; doi:10.1371/journal.pone.0077478)

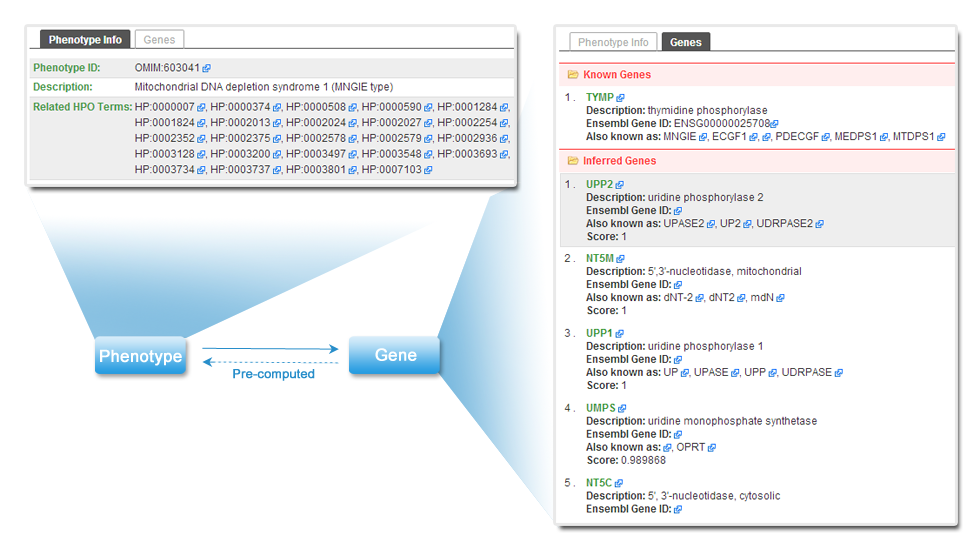

Supplement: Figure S1 — Workflow and contents when accessing the web server from phenotypes. The candidate genes of the query phenotypes are retrieved from the pre-computed gene-phenotype associations. (TIF). (TIF) [file pone.0077478.s001.tif]

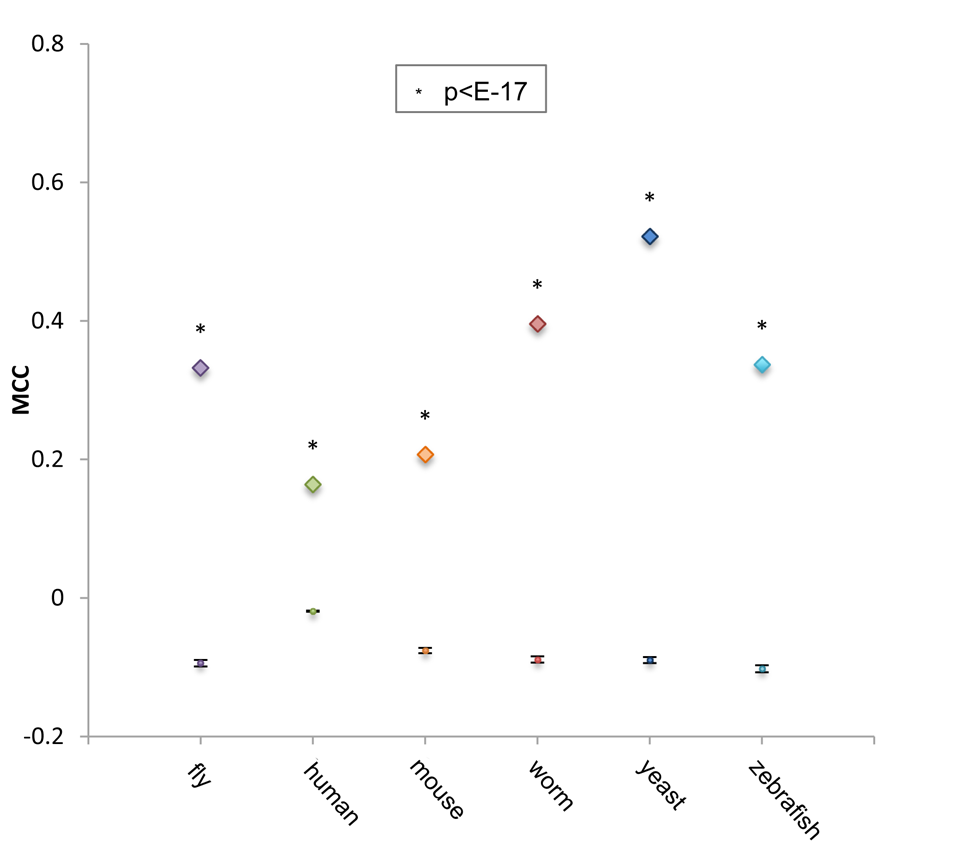

Supplement: Figure S2 — MCC of our prediction and randomization for each species. The MCC values for the prediction and 100-time randomization. (TIF). (TIF) [file pone.0077478.s002.tif]
