# Supplementary material for: Inference of Gene-Phenotype Associations via Protein-Protein Interaction and Orthology
Source: PLoS One. 2013 Oct 23;8(10):e77478. doi: 10.1371/journal.pone.0077478 (PMC3806783; doi:10.1371/journal.pone.0077478)
Supplement: Table S1 — Phenotype and known gene-phenotype association sources. The sources from which the phenotype data and known gene-phenotype associations were retrieved are listed. (TXT). (DOCX) [file pone.0077478.s003.docx]

### Table S1

**Table S1. Phenotype and known gene-phenotype association sources.**

| **Species** | **Phenotype** | **Known gene-phenotype association** |
| --- | --- | --- |
| Fly | fly_anatomy.obo, flybase_controlled_vocabulary.obo, flybase_stock_vocabulary.obo, fly_development.obo, gene_ontology.obo, image.obo,psi-mi.obo, so.obo (<http://www.obofoundry.org>) | Allele_phenotypic_data_fb_2012_06.tsv (<http://flybase.org>) |
| Human | omim.txt (<http://omim.org>), human-phenotype-ontology.obo (<http://www.obofoundry.org>) | morbidmap ([http://omim.org](http://omim.org/)), phenotype_annotation.omim (<http://www.human-phenotype-ontology.org>) |
| Mouse | mammalian_phenotype.obo (<http://www.obofoundry.org>) | MGI_PhenoGenoMP.rpt ([http://informatics.jax.org](http://informatics.jax.org/)) |
| Worm | worm_phenotype.obo (<http://www.obofoundry.org>) | phenotype_association.WS234.wb (<http://www.wormbase.org>) |
| Yeast | ascomycete_phenotype.obo (<http://www.obofoundry.org>) | Phenotype_data.tab at ([http://yeastgenome.org](http://yeastgenome.org/)) |
| Zebrafish | zebrafish_anatomy.obo (<http://www.obofoundry.org>) | Phenotype.txt ([http://zfin.org](http://zfin.org/)) |
