# Supplementary material for: Inference of Gene-Phenotype Associations via Protein-Protein Interaction and Orthology
Source: PLoS One. 2013 Oct 23;8(10):e77478. doi: 10.1371/journal.pone.0077478 (PMC3806783; doi:10.1371/journal.pone.0077478)
Supplement: Table S2 — The average AUC values of different λ for the six species. (TXT). (DOCX) [file pone.0077478.s004.docx]

### Table S2

**Table S2 The average AUC values of different *λ* for all species.**

| ***λ*** | **Fly** | **Human** | **Mouse** | **Worm** | **Yeast** | **Zebrafish** |
| --- | --- | --- | --- | --- | --- | --- |
| 0 | 0.756 | 0.213 | 0.589 | 0.723 | 0.727 | 0.727 |
| 0.1 | 0.788 | 0.606 | 0.688 | 0.712 | 0.810 | 0.692 |
| 0.2 | 0.798 | 0.620 | 0.693 | 0.733 | 0.819 | 0.724 |
| 0.3 | 0.806 | 0.639 | 0.698 | 0.745 | 0.828 | 0.740 |
| 0.4 | 0.813 | 0.656 | 0.701 | 0.752 | 0.836 | 0.742 |
| 0.5 | 0.821 | 0.688 | 0.707 | 0.760 | 0.844 | 0.756 |
| 0.6 | 0.828 | 0.704 | 0.711 | 0.769 | 0.851 | 0.755 |
| 0.7 | 0.833 | 0.713 | 0.716 | 0.777 | 0.857 | 0.762 |
| 0.8 | 0.838 | 0.714 | 0.717 | 0.778 | 0.863 | 0.765 |
| 0.9 | 0.840 | 0.712 | 0.7181 | 0.777 | 0.868 | 0.761 |
| 1 | 0.824 | 0.693 | 0.709 | 0.736 | 0.864 | 0.654 |
