# Supplementary material for: Inference of Gene-Phenotype Associations via Protein-Protein Interaction and Orthology
Source: PLoS One. 2013 Oct 23;8(10):e77478. doi: 10.1371/journal.pone.0077478 (PMC3806783; doi:10.1371/journal.pone.0077478)
Supplement: Table S3 — The most enriched GO terms for mouse phenotype insulin resistance (MP: 0005331). (TXT). (DOCX) [file pone.0077478.s005.docx]

### Table S3

**Table S3 The most enriched GO terms for mouse phenotype insulin resistance (MP: 0005331).**

| **Phenotype** | **P value** | **GO term** |
| --- | --- | --- |
| Insulin resistance | 7.79E-08 | Lipid metabolic process (GO:0006629) |
|  | 1.46E-07 | Palmitoyl-CoA hydrolase activity (GO:0016290) |
|  | 4.09E-07 | Acyl-CoA hydrolase activity (GO:0047617) |
|  | 5.20E-07 | Carboxylesterase activity (GO:0004091) |
|  | 5.72E-07 | Catalytic activity (GO:0003824) |
|  | 9.53E-07 | Hydrolase activity (GO:0016787) |
|  | 1.80E-06 | Hydrolase activity, acting on ester bonds (GO:0016788) |
|  | 1.91E-06 | Metabolic process (GO:0008152) |
|  | 1.59E-05 | Carboxylic acid metabolic process (GO:0019752) |
|  | 2.57E-05 | Response to peptide hormone stimulus (GO:0043434) |
